# Mycobacterium intracellulare-Related Immune Reconstitution Syndrome in an HIV Patient: A Case Report

**DOI:** 10.7759/cureus.22995

**Published:** 2022-03-09

**Authors:** Sebastian Quintero Montealegre, Natalia Medina Jimenez, Diego Molina Castro

**Affiliations:** 1 Internal Medicine, Pontifical Xavierian University, Bogotá, COL; 2 HIV/AIDS, Sociedad Integral de Especialistas en Salud, Medellín, COL; 3 Internal Medicine, Hospital San Vicente Fundación, Medellín, COL

**Keywords:** non-tuberculous, cervical lymphadenopathy, iris, aids, hiv

## Abstract

Immune reconstitution syndrome (IRIS) is an uncommon complication seen in patients living with human immunodeficiency virus (HIV) characterized by the decline in a pre-existing condition or a new diagnosis of a masked disease. It is associated with a significant inflammatory component that occurs after starting antiretrovirals, being most frequent in those with severe immunosuppression. Thereby, different types of opportunistic diseases such as non-tuberculous mycobacteria are more frequent in this group of patients, especially those with low lymphocyte counts of below 50 cells/µL. Here, we present the case of an HIV-positive patient who developed lymphadenitis caused by *Mycobacterium intracellulare* as an unmasked IRIS after initiating treatment for HIV.

## Introduction

It is well known that highly active antiretroviral therapy (HAART) leads to immunovirological control in the treatment of human immunodeficiency virus (HIV) infection, decreasing the frequency of opportunistic diseases and improving survival. However, it is associated with different complications such as the immune reconstitution syndrome (IRIS). IRIS was first described as being related to an increase in localized non-tuberculous mycobacteria after the introduction of treatment with zidovudine in the early 1990s [1].

Here, we present the case of an HIV-positive patient who developed lymphadenitis caused by *Mycobacterium intracellulare* as an unmasked IRIS after initiating treatment for HIV.

## Case presentation

A 22-year-old heterosexual male with a history of daily tetrahydrocannabinol use was diagnosed with HIV in 2019. He was lost to follow-up after the first medical visit but returned in May 2020 when he decided to start HAART with tenofovir disoproxil 300 mg + efavirenz 600 mg + emtricitabine 200 mg; suspending it three days later because of marked intolerance with no reintroduction after this episode. Later, he was hospitalized in June 2020 with a new diagnosis of meningeal cryptococcosis treated with amphotericin B and fluconazole. In the July 2020 follow-up, he initiated abacavir 600 mg + lamivudine 300 mg + dolutegravir 50 mg with good adherence and tolerance, achieving undetectable viral load (Table 1).

**Table 1:** Immunovirology evolution. HIV: human immunodeficiency virus; HAART: highly active antiretroviral therapy

	Diagnosis	Start of treatment	First control after starting HAART
HIV viral load	85 × 10^4 ^copies/mL (log 5.92)	105 × 10^4^ copies/mL (log 6.02)	40 copies/mL (log 1.6)
CD4 T lymphocytes	25 cells/µL (2.79%)	15 cells/µL (2.38%)	127 cells/µL (10.57%)
CD8 T lymphocytes	457 cells/µL (50.74%)	229 cells/µL (37.1%)	552 cells/µL (45.78%)
CD3 T lymphocytes	532 cells/µL (59.11%)	275 cells/µL (44.61%)	751 cells/µL (62.28%)
Ratio CD4/CD8	0.06	0.06	0.23

**Figure 1:**
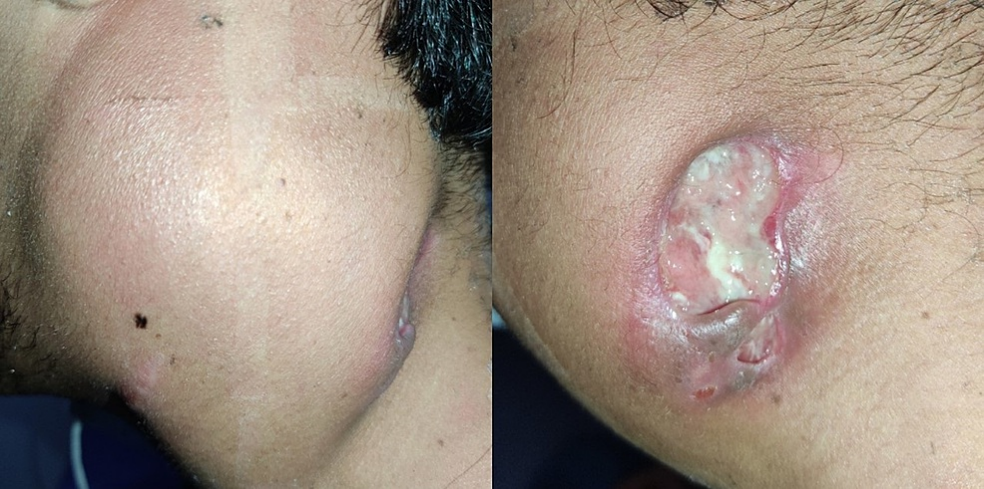
Left cervical mass with ulceration.

In November 2020, he required admission to a hospital for a month due to a left cervical mass with tenderness and an ulcer with active suppuration (Figure 1). Blood test showed creatinine 0.92 mg/dL, blood urea nitrogen 15.74 mg/dL, erythrocyte sedimentation rate 53 mm/hour, white blood cell count 8,910 cell/mL, neutrophils 72.7%, lymphocytes 14%, eosinophils 2%, hemoglobin 13.4 g/dL, platelets 474,000 cells/mL, aspartate aminotransferase 17.3 U/L, alanine aminotransferase 17.5 U/L, alkaline phosphatase 107.2 U/L, and total bilirubin 0.42 mg/dL.

**Figure 2:**
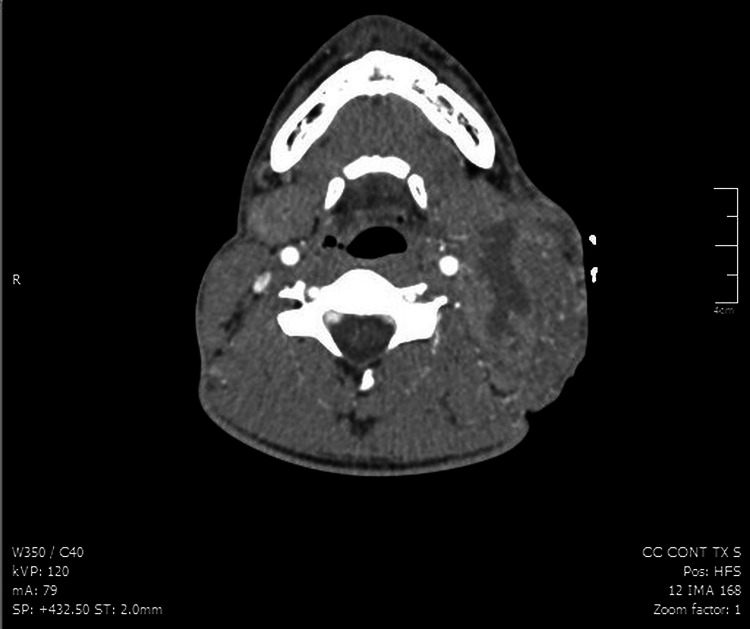
Neck CT showing a left lymph node conglomerate with hypodense images due to necrotic changes, loss of cleavage plane with muscle structures, and mass effect on the ipsilateral jugular vein. CT: computed tomography

A contrast computed tomography (CT) of the neck revealed a left lymphadenopathy conglomerate with central hypodensity and loss of the cleavage plane with the adjacent muscular structures (Figure 2). Chest projection showed small mediastinal and axillary lymphadenopathies with no involvement of the lung parenchyma.

A fine-needle aspiration biopsy was performed. The results showed negative Gram staining and bacterial culture. The acid-fast bacilli smear was positive (++), but the polymerase chain reaction assay GeneXpert MTB/RIF was negative. The patient started antituberculous treatment (rifampicin 600 mg qd + isoniazid 300 mg qd + pyrazinamide 1,200 mg qd + ethambutol 1,100 mg qd), prednisone 40 mg qd for two weeks, and an adjustment of dolutegravir dosage (50 mg bid) due to the possible interactions with rifampicin. During the next few months, there was no clinical response, despite the fact that the patient was adherent to his treatment, and drug interactions were ruled out, triggering the possibility of non-tuberculous mycobacteria. This was confirmed by the definitive culture results and a new molecular test (GenoType) isolating *Mycobacterium intracellulare* with an absence of *rrl *and *rrn *gene mutations (macrolide and aminoglycoside sensibility). With these new findings, the treatment was switched to rifampicin 600 mg qd + ethambutol 1,200 mg qd + azithromycin 500 mg qd, and no further use of steroids was necessary. Rapid response was seen within a month during the follow-up visit.

## Discussion

Multiple infectious agents and pathological processes are related to IRIS (Table 2), hindering the efforts for a universal definition. However, there is a temporal association between the initiation of HAART and the onset of symptoms, with patients presenting with a reduction in the HIV viral load and a rise in the CD4 lymphocyte count, the latter not being a necessary condition [2].

**Table 2:** Infectious etiologies related to immune reconstitution syndrome. Adapted from: Walker NF, Scriven J, Meintjes G, Wilkinson RJ: Immune reconstitution inflammatory syndrome in HIV-infected patients. HIV AIDS (Auckl). 2015, 7:49-64. 10.2147/HIV.S42328 [4].

	Names of organisms
Bacteria	*Mycobacterium tuberculosis*, *Mycobacterium avium-intracellulare*, *Mycobacterium genavense*, *Mycobacterium kansasii*, *Mycobacterium scrofulaceum*, *Mycobacterium xenopi*, *Bartonella *spp., *Chlamydia trachomatis*
Virus	Herpes simplex 1 and 2, varicella-zoster, cytomegalovirus, Epstein–Barr, hepatitis B and C, polyomavirus
Fungi	*Cryptococcus neoformans*, *Pneumocystis jirovecii*, *Histoplasma *spp., *Candida *spp., *Tinea corporis*
Parasites	*Toxoplasma gondii*, *Schistosoma mansoni*, *Leishmania *spp., *Leishmania infantum*, *Leishmania braziliensis*, *Strongyloides stercoralis*, *Cryptosporidium *spp., *Microsporidium *spp.

In a meta-analysis involving a total of 13,103 patients from 54 studies, Muller et al. found IRIS incidence at an average of 16.1% with a mortality of 4.5%. They also identified a difference in the etiologies: 37.7% cytomegalovirus retinitis, 19.5% meningeal cryptococcosis, 16.7% progressive multifocal leukoencephalopathy, 15.7% tuberculosis, and 6.4% Kaposi sarcoma [3].

Despite the heterogeneity of this syndrome, some risk factors have been described that help the identification of these patients: a rapid decline in the viral load (in the first three months), an initial CD4 count lower than 50 cells/µL, early initiation of HAART in patients with the concomitant opportunistic disease, and no previous HAART exposure [2]. IRIS has the following two patterns classified by the clinical presentation: paradoxical IRIS, wherein symptoms and signs of an opportunistic infection briefly improve but reappear or worsen despite treatment, and unmasked IRIS, wherein a new opportunistic infection not previously diagnosed appears after HAART with excessive inflammation [4].

Different infectious etiologies related to IRIS has been described such as *Mycobacterium intracellulare*, a slow-growing and ubiquitous microorganism in the environment belonging to the *Mycobacterium avium* complex (MAC), which typically affects individuals with HIV in advanced stages of the disease, especially with CD4 lymphocyte counts less than 50 cells/µL [5]. In these patients, both paradoxical and unmasked IRIS have been reported, presenting atypically with high focal inflammation, the most frequent manifestations being peripheral lymphadenitis, thoracic-pulmonary, and intra-abdominal disease [6].

Unlike *Mycobacterium tuberculosis* infection, non-tuberculous mycobacterial (NTM) infections are not mandatory to report, hindering the understanding of epidemiological behavior. Henkle et al. identified 334 patients with extrapulmonary NTM infection from 2007 to 2012. In their study, 8.4% of the patients had lymph node involvement. The authors reported an annual incidence of 0.7 cases of *Mycobacterium avium*/*intracellulare *complex (MAC) for 100,000 habitants [7]. In Colombia, Montúfar et al. analyzed 187 patients with mycobacterial infection identified by culture, finding NTM in 9.1% of cases, of which, 35.29% of the cases had MAC infection and 41.17% had HIV infection, with only one patient presenting lymph node involvement [8].

The onset of symptoms is usually slow, with patients initially presenting with constitutional symptoms (fever, night sweats, weight loss, abdominal pain, diarrhea, and malaise), which may be absent or can be associated with anemia, hepatosplenomegaly, and lymphadenopathy. Regarding lymphadenitis, the growth of the lymph nodes begins painlessly, evolving with the production of pus [9]. Imaging findings are usually non-specific: solid and hypoechoic nodes are identified on ultrasound or hypodense with a necrotic center on CT [10].

Lymphadenitis diagnosis is based on cultures (positivity range from 50% to 80%), molecular test, or histopathological findings, typically caseating granulomas. The presence of acid-alcohol-resistant bacilli is variable, and infection by *Mycobacterium tuberculosis* must be ruled out [11].

Treatment is based on a macrolide plus ethambutol and/or rifampicin with a duration ranging between six to twelve months, depending on clinical evolution and sometimes requiring surgical management. MAC-related IRIS in HIV patients is based on surgical and antimicrobial management, the continuation of antiretroviral therapy, and the use of systemic corticosteroids, generally when it presents with painful lymphadenopathy [12].

## Conclusions

IRIS is a condition that must be considered at the time of antiretroviral therapy initiation, especially in patients with marked immunosuppression. Despite not being a frequent disease, non-tuberculous mycobacterial infections should not be forgotten, especially when molecular identification tests for tuberculosis are negative.
